# MTFP1 is a mitophagy receptor that operates in PINK1/PRKN-dependent mitophagy and promotes oral cancer cell survival

**DOI:** 10.1080/27694127.2023.2267882

**Published:** 2023-10-16

**Authors:** Debasna P Panigrahi, Sujit K Bhutia

**Affiliations:** Cancer and Cell Death Laboratory, Department of Life Science, National Institute of Technology Rourkela, Odisha, India

**Keywords:** Apoptosis, MTFP1, Mitophagy, Mitochondrial fission, PRKN

## Abstract

MTFP1 (mitochondrial fission process 1), an inner mitochondrial membrane protein, plays a crucial role in mitochondrial fission to maintain mitochondrial morphology. Our study found that MTFP1 contains a LIR (LC3-interacting region) to interact with MAP1LC3B (microtubule-associated protein 1 light chain 3 beta) and serves as a mitophagy receptor to eliminate damaged mitochondria. Interestingly, mutation of MTFP1 LIR motif (MTFP1mLIR) inhibits this interaction, decreasing mitophagy in oral cancer cells. Moreover, knockdown of PRKN (parkin RBR E3 ubiquitin protein ligase) or PINK1 (PTEN-induced kinase 1) abolished mitophagy in MTFP1-overexpressing oral cancer cells. In this setting, we observed that MTFP1mLIR-expressing cells display a decrease in TOMM20 (translocase of outer mitochondrial membrane 20) levels without affecting those of COX4 (cytochrome c oxidase subunit 4). In contrast, loss of PRKN or PINK1 caused inhibition of both TOMM20 and COX4 degradation in MTFP1mLIR-expressing cells exposed to cellular stress, suggesting that PRKN may activate the rupture of outer mitochondrial membrane in MTFP1-overexpressing cells for effective mitophagy. We also observed that MTFP1 is beneficial to oral cancer cell survival exposed to anticancer drugs, such as cisplatin, through mitophagy, since inhibition of MTFP1-dependent mitophagy induced cell death. Thus, targeting MTFP1-associated mitophagy could represent a strategy for oral cancer therapy.

**Abbreviations:** BBC3/PUMA, BCL2 binding component 3; BCL2L13, BCL2 like 13; BINIP3L, BCL2 interacting protein 3 like; BNIP3, BCL2 interacting protein 3; CCCP, Carbonyl cyanide m-chlorophenylhydrazone; COX4, cytochrome c oxidase subunit 4; DNM1L, dynamin 1 like;FKBP8, FKBP prolyl isomerase 8; FIS1, fission, mitochondrial 1; FUNDC1, FUN14 domain containing 1; LIR, LC3 interacting region; MTFP1, mitochondrial fission process 1; PHB2, prohibitin 2; PI3K, Phosphatidylinositol 3-kinase; PRKN, Parkin RBR E3 ubiquitin protein ligase; PINK1, PTEN induced kinase 1; TOMM20, translocase of outer mitochondrial membrane 20

Mitochondria are the powerhouse of the cell and regulate cellular homeostasis, metabolism, morphology and cell fate. To maintain mitochondria function, damaged mitochondria are cleared by a cellular process known as mitophagy. Several mitophagy receptors, including BNIP3 (BCL2 interacting protein 3), BINIP3L (BCL2 interacting protein 3 like), FUNDC1 (FUN14 domain containing 1), BCL2L13 (BCL2 like 13), BBC3/PUMA (BCL2 binding component 3), FKBP8 (FKBP prolyl isomerase 8), and PHB2 (prohibitin 2) regulate mitophagy in response to different cellular stresses. MTFP1 (mitochondrial fission process 1) is mainly localized to the inner mitochondrial membrane and has an essential role in mitochondrial fission. Previous studies reported that MTFP1 is a novel human nuclear-encoded protein with a molecular mass of 18 kDa and three transmembrane domains. Interestingly, the C-terminus of MTFP1, which contains two transmembrane domains, is essential for both targeting this protein to the mitochondrial inner membrane and fission activity. In addition, MTFP1 is a downstream effector of the class I phosphatidylinositol 3-kinase (PI3K) signalling, which is key to maintain mitochondrial morphology and cell survival. In our recent study, we initially analyzed mitochondrial fission in oral cancer cells and found that MTFP1 knockdown diminishes the levels of the mitochondrial fission protein FIS1 (fission, mitochondrial 1) as well as phosphorylation of DNM1L (dynamin 1 like) at Ser616, which indicates a mitochondrial hyperfusion. Furthermore, MTFP1-depleted cells showed elongated mitochondria that form a more extensive mitochondrial network than in control cells, confirming that MTFP1 controls mitochondrial fission in oral cancer cells.^1^

Our study also identified MTFP1 as a mitophagy receptor, which participates in mitophagy. Mitophay was assessed by quantifying the degradation of the outer mitochondrial membrane protein TOMM20 (translocase of outer mitochondrial membrane 20) and the inner mitochondrial membrane protein COX4 (cytochrome c oxidase subunit 4). To understand the connection between MTFP1-induced mitochondrial fission and mitophagy in oral cancer cells, we studied mitophagy in DNM1L deficient cells expressing MTFP1 and found that MTFP1 controls mitochondrial fission during CCCP (carbonyl cyanide m-chlorophenylhydrazone)-induced mitophagy. Thus, we hypothesized that MTFP1 is a new mitophagy receptors. Autophagy receptors often possess a LIR (LC3 interacting region) to promote the sequestration of specific cargoes into autophagosomes by interacting with memmebr of the ATG8 protein family. Utilizing the iLIR *in silico* database, we have identified three putative LIR motifs in MTFP1 between amino acids 32–35, 87–90 and 157–160. Two of them, the ones between amino acids 32–35 and 87–90, are in the transmembrane segments of MTFP1, and therefore they very likely cannot participate in mitophagy. So, we hypothesized that the Tyr157, Pro158 Thr159, and Val160 residues of MTFP1 might function as a LIR motif in the interaction of MTFP1 with members of the LC3 protein family. To confirm this, we generated a mutant that we named MTFP1mLIR, and carries the Y157A and V160A changes and overexpressed it in FaDu cells before carrying out immunoprecipitation and colocalization experiments. These experiments revealed that, opposite to the wild-type protein, MTFP1mLIR does not bind to MAP1LC3B (microtubule associated protein 1 light chain 3 beta) and cannot sustain CCCP-induced mitophagy, although it still functions in mitochondrial fission.

MTFP1 is an inner mitochondrial membrane protein. As a result, the rupture of the outer mitochondrial membrane must be essential for MTFP1 to bind with MAP1LC3B and mediate mitophagy. To verify this scenario, we studied CCCP-induced mitophagy in the presence of the proteasomal inhibitor epoximicin in MTFP1-overexpressing cells and observed that epoximicin blocked the colocalization of MTFP1 and MAP1LC3B, and counteracted the decrease in the level of COX4. This observation suggested that proteasomal degradation may be involved in rupturing the outer mitochondrial membrane during mitophagy. PRKN (Parkin RBR E3 ubiquitin protein ligase) is a cytosolic E3 ubiquitin ligase that translocates to mitochondria to ubiquitinate several candidate substrate proteins on the surface of mitochondria. PINK1 (PTEN induced kinase 1) has been recognized for sensing damaged mitochondria associated with PRKN recruitment to the mitochondria for proteasomal degradation and is also involved in specific types of autophagy like the ones induced by mitochiondrial depolarization. To examine the role of PRKN in MFTP1-mediated mitophagy, we knocked down PRKN in MFTP1-overexpressing cells, and showed that loss of PRKN inhibits CCCP-induced mitophagy in oral cancer cells. Similarly, a siRNA targeting PINK1 was transiently transfected into MTFP1-overexpressing cells, and siPINK1 also resulted in a block of MTFP1-induced mitophagy. Interestingly, PRKN or PINK1 deficiency caused inhibition of both TOMM20 and COX4 degradation in MTFP1mLIR-overexpressing cells, but a decrease in TOMM20 level without affecting those of COX4 level was observed only in the MTFP1mLIR-expressing cells, confirming that PRKN is vital for degrading the outer mitochondrial membrane and not for mitophagy in CCCP-induced mitophagy in MTFP1-overexpressing oral cancer cells.

Finally, we explored whether MTFP1 is beneficial for the survival of oral cancer cells exposed to anticancer drugs such as cisplatin. Thus, we examined cell death programs in MTFP1-overexpressing oral cancer cells treated with cisplatin in the presence of wortmannin, a PI3K inhibitor, and Mdivi1, a DNM1L inhibitor that also blocks MTFP1-induced mitophagy. Interestingly, we found that mitophagy inhibition in MTFP1-overexpressing cells significantly decreased cell viability and increased apoptosis upon exposure to cisplatin in comparison to the control MTFP1-overexpressing cells, suggesting that MTFP1 may enhance survival by preventing cell death through mitophagy in oral cancer cells.

The highlight of our work is that MTFP1 serves as a mitophagy receptor to execute mitophagy (Fig.1). Moreover, MTFP1-mediated mitophagy provide a survival benefit to oral cancer cells, and MTFP1 deficiency triggers apoptosis, suggesting MTFP1 controls cell survival through mitochondrial fission and/or mitophagy in oral cancer cells. Thus, targeting MTFP1 could be a potential therapeutic strategy for treating oral cancer and possibly other malignancies.

**Figure 1.**
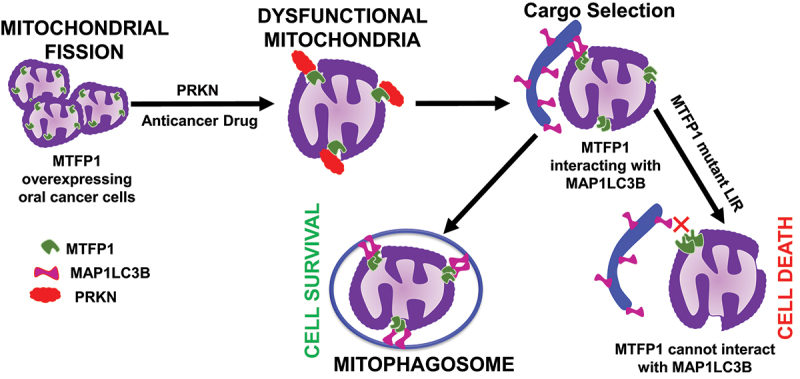
In oral cancer cells, MTFP1 plays a crucial role in mitochondrial fission and mitochondrial morphology maintenance. Upon exposure to anticancer drugs, MTFP1 interacts with MAP1LC3B, promoting mitophagy to eliminate dysfunctional mitochondria through PINK1/PRKN-dependent mitophagy. MTFP1-dependent mitophagy is cytoprotective and provides survival benefits to cancer cells. The mutant LIR MTFP1 can not interact with MAP1LC3B to induce mitophagy, leading to cell death during anticancer treatment.

## References

[1] Panigrahi DP, Praharaj PP, Behera BP, et al. The inner mitochondrial membrane fission protein MTP18 serves as a mitophagy receptor to prevent apoptosis in oral cancer. J Cell Sci. 2023 Jul 1;136(13):jcs259986. doi: 10.1242/jcs.259986. 37313742

